# Microbial Composition of Oral Biofilms after Visible Light and Water-Filtered Infrared a Radiation (VIS+wIRA) in Combination with Indocyanine Green (ICG) as Photosensitizer

**DOI:** 10.3390/antibiotics9090532

**Published:** 2020-08-23

**Authors:** Thomas Burchard, Lamprini Karygianni, Elmar Hellwig, Annette Wittmer, Ali Al-Ahmad

**Affiliations:** 1 Department of Prosthetic Dentistry, Center for Dental Medicine, Medical Center, University of Freiburg, Faculty of Medicine, University of Freiburg, 79085 Freiburg, Germany; thomas.burchard@uniklinik-freiburg.de; 2 Clinic of Conservative and Preventive Dentistry, Center of Dental Medicine, University of Zürich, 8006 Zürich, Switzerland; Lamprini.Karygianni@zzm.uzh.ch; 3 Department of Operative Dentistry & Periodontology, Center for Dental Medicine, Medical Center, University of Freiburg, Faculty of Medicine, University of Freiburg, 79085 Freiburg, Germany; elmar.hellwig@uniklinik-freiburg.de; 4 Department of Microbiology and Hygiene, Institute of Medical Microbiology and Hygiene, Medical Center, University of Freiburg, 79085 Freiburg, Germany; annette.wittmer@uniklinik-freiburg.de

**Keywords:** antimicrobial photodynamic therapy (aPDT), indocyanine green (ICG), oral biofilm, visible light and water-filtered infrared A (VIS+wIRA)

## Abstract

In view of increasing antibiotic resistance, antimicrobial photodynamic therapy (aPDT) is an alternative treatment method used to eradicate the microbial community of oral biofilms that can be responsible for different oral infections. In order to investigate changes in the microbial composition after application of aPDT with visible light and water-filtered infrared A (VIS+wIRA) in combination with indocyanine green (ICG), oral microorganisms of the initial and mature biofilm were evaluated by mass spectrometry (MALDI-TOF-MS). To determine surviving microorganisms using MALDI-TOF-MS, an in situ biofilm was irradiated with VIS+wIRA for five minutes in the presence of ICG (300 and 450 µg/mL, respectively). Treatment with chlorhexidine (0.2%) served as positive control. Identified microorganisms of the initial biofilm treated with ICG showed a clear reduction in diversity. The microbial composition of the mature oral biofilm also showed changes after the implementation of aPDT, which mainly resulted in a shift in the percentage of bacterial species. The resulting destruction of the microbial balance within the oral biofilm by aPDT using VIS+wIRA and ICG can be seen as an advantageous supplementary approach in the adjunctive treatment of periodontitis and peri-implantitis.

## 1. Introduction

The treatment of biofilm-associated diseases is one of the most important challenges in medicine, especially due to increasing antibiotic resistance. Consisting of different bacterial species and extracellular polymeric substance, biofilms can be found in many border areas in the human body and can cause serious infections in the ears, urinary tract, around catheters or implants and especially in the oral cavity [1]. More than 60% of all microbial infections seem to be related to microbial biofilms [2]. This also includes oral biofilms consisting of more than 700 different microorganisms, of which not all are known to date [3,4,5]. Numerous bacteria colonize the oral cavity from birth, considered to be physiological [6]. The question of “which bacterial composition is defined as healthy oral flora” still remains open. In this context, diverse literature reports describe the health-promoting properties of individual microorganisms, such as *Actinomyces* spp., *Neisseria* spp., *Granulicatella* spp., *Veillonella* spp. and some streptococci [7]. Zaura et al. (2009) investigated the microbial composition of healthy oral cavities with the aim of identifying a core microbiome that is said to be responsible for the development of functional stability and homeostasis [8]. The study showed a large number of matching bacterial sequences in unrelated subjects, a fact which supports the theory of a basic core microbiome [8]. The predominant phyla found in the study were Firmicutes (genera *Streptococcus, Granulicatella* and *Veillonella*), Actinobacteria (genera *Actinomyces*, *Rothia* and *Corynebacterium*), Proteobacteria (genera *Neisseria* and *Haemophilus*), Fusobacteria and Bacteroidetes (genera *Prevotella, Porphyromonas* and *Capnocytophaga*).

However, oral bacteria also have the potential to trigger serious diseases [3]. Pathogens, such as *Aggregatibacter actinomycetemcomitans* or *Porphyromonas gingivalis*, which belong to the group of Gram-negative bacteria, are suspected of causing periodontal diseases [7,9,10]. With regard to the problematic formation of dental plaque and caries, Gram-positive cocci and rods are often involved. These Gram-positive cocci include bacterial species such as *Streptococcus mutans*, *Streptococcus mitis* and *Streptococcus oralis*, while the Gram-positive rods include bacterial species such as lactobacilli or representatives of *Actinomyces* spp. [6,11]. In addition to causing periodontal infections, the subgingival biofilm can also be involved in the development of systemic diseases [12]. Despite the developments of chemical agents for microbial inactivation, the concern of microorganisms developing resistance within the biofilm remains [13]. It is therefore necessary to find further supportive therapeutic approaches that specifically destroy the balance of microorganisms in the biofilm, as this is responsible for microbial resistance [14].

In contrast to the unfavorable results of conventional oral biofilm treatments, the application of antimicrobial photodynamic therapy (aPDT) as a non-invasive, biofilm-specific photochemical technique promises a cost-effective treatment of oral infections and can be considered as an adjunctive treatment method to antibiotic therapy [15,16]. For aPDT, a photosensitizing substance, the presence of oxygen and a suitable light source with a wavelength complying with the absorption spectrum of the photosensitizer are required in order to exert an unspecific damaging effect [17]. Upon illumination, the photosensitizer absorbs radiation energy, which leads to the excitation of its state and results, via stable intermediate steps, in the generation of reactive oxygen species (ROS) [13]. Conversely, part of the excited photosensitizer remains unstable and does not carry out any subsequent reactions, but releases its energy as fluorescence or heat [18]. Common broadband halogen lamps or light-emitting diodes (LED) are suitable as light sources for the purpose of light-induced activation [13,19,20,21]. Nevertheless, the limited emission wavelength spectrum associated with low-cost LED devices and halogen lamps leads to tissue overheating [22].

Protection of an irradiated structure can be accomplished by the already described broadband light source, consisting of a combination of visible light wavelengths (VIS) with water-filtered infrared A (wIRA) radiation [23]. The water filter reduces the main proportions of infrared B and infrared C, so that the radiation is limited to the wavelength range of VIS and wIRA (VIS+wIRA) [24]. In contrast to conventional halogen lamps, radiators emitting wIRA enable higher energy transfer into the tissue and reduce the thermal load on superficial tissue layers at the same time [25]. Additional advantages can be seen in an increased partial pressure of oxygen in the tissue and an improved blood flow to the tissue, which promotes tissue regeneration and pain relief [26,27].

Numerous studies have already reported high rates of reduction in viable microorganisms by aPDT of oral biofilms using VIS+wIRA in combination with photosensitizers such as toluidine blue (TB) and chlorine e6 (Ce6) [23,28]. Similar promising results regarding the number of surviving bacteria were also achieved in a recent study with the photosensitizer indocyanine green (ICG) [29]. This anionic photosensitizer is characterized by low toxicity, good tolerance and rare occurrence of side effects [30]. Based on many years of use as a diagnostic agent, for example, for examining liver function, in the field of ophthalmology and in dermatology, in which the photosensitizer was injected intravenously for examining circulatory conditions, a safe use of ICG can be considered [31,32]. The high affinity to bind to proteins and rapid elimination via the liver prevent accumulation in the body and consequently repeated applications of the photosensitizer are possible [30]. However, it is still unknown whether the microbial population in the biofilm treated with ICG remains pathogenic, and further biofilm formation is to be expected.

For this reason, the current study examined the influence of aPDT with VIS+wIRA in combination with ICG on the in situ microbial community after a biofilm formation period of two hours and three days, respectively. The composition of surviving microorganisms from the treated initial and mature oral biofilms was determined by the culture technique. The obtained results serve as a prerequisite for further clinical studies to gain knowledge on the possible use of ICG as a photosensitizer with VIS+wIRA as an adjunctive to conventional methods in the treatment of periodontal infections in dentistry.

## 2. Materials and Methods

### 2.1. Photosensitizer and Light Source

In contrast to many previous studies on aPDT, the photosensitizer ICG (perio green^®^, elexxion, Singen, Germany) was used. The optical absorption spectrum of ICG showed maximum absorption peaks in the range from 800 to 830 nm [33]. Due to the contraindication given by the manufacturer regarding iodine allergy, it can be assumed that about 5% iodide is added to improve the solubility of the photosensitizer [34]. For the preparation of the ICG solution, the substance had to be dissolved with water (Aqua ad iniectabilia Braun, B. Braun, Melsungen, Germany) as a solvent in concentrations of 300 and 450 µg/mL ICG [32]. The dissolved ICG was stored in the dark for no longer than four hours in order to exclude premature light-induced photochemical reactions and to prevent a reduced effect of the photosensitizer [35].

Since the binding to plasma proteins leads to a concentration-dependent shift in the absorption spectrum of ICG, a light source with a broad wavelength spectrum was chosen [36]. For the purpose of light-induced excitation, a VIS+wIRA broadband radiator (Hydrosun^®^ 750 FS, Hydrosun Medizintechnik, Müllheim, Germany) with a 7 mm water cuvette was used, as described in other studies [23,37,38,39]. Instead of the standard light filter, an orange filter of type BTE 31 was investigated, which emphasizes the desired emission in relation to the absorption spectrum of protoporphyrin IX of more than twice the conventional filter (BTE 595). Due to the absorption by the water molecules, there is a continuous wavelength spectrum from 570 to 1400 nm with local minima at 970, 1200 and 1430 nm [40]. The radiator was set up at a distance of 20 cm from the specimens prepared and deposited in a water bath. For all irradiated test groups, an effective irradiation with VIS+wIRA of 200 mW/cm^2^ was applied, which consisted of approximately 48 mW/cm^2^ VIS and 152 mW/cm^2^ wIRA [23]. Emissions of ultraviolet radiation as well as infrared B and C, which were emitted by the halogen lamp (dimensions: 28 × 27 × 28 cm) with a power consumption of 750 W (nominal voltage: 230 V, 50–60 Hz), were negligible due to absorption by the water filter [41].

### 2.2. Sample Preparation and Biofilm Formation

For the preparation of the specimens, incisors from 2-year-old BSE-free cattle that were slaughtered at a slaughterhouse were used (Emil Färber GmbH & Co. KG, Freiburg, Germany). In the manufacture of the cylindrical specimens (diameter: 5 mm, surface: 19.63 mm^2^, height: 1.5 mm), only the tooth surfaces facing the cheek were used, as has already been described in other studies [42]. The bovine enamel slabs (BES) were polished using a wet grinder (Knuth-Rotor-3, Struers, Willich, Germany) using sandpaper (grit sizes from 250 to 4000) with decreasing grain size. Finally, the specimens were cleaned according to a disinfection protocol as described in earlier studies [43]. With the help of a situation model of the upper jaw, an individually adapted maxillary splint made of polymethylmethacrylate (PMMA) was manufactured for each of the study participants, to whose side wings six BES were attached using an addition-curing silicone (Panasil^®^ initial contact X-Light, Kettenbach, Eschenburg, Germany) [28]. The maxillary splint enabled precise positioning of the BES in the interdental area facing the cheek between the premolars and molars and prevented movements of the tongue or cheek from causing disturbances in biofilm formation during the study period [37].

A total of 12 BES were worn for a duration of two hours or three days by each of the healthy study participants. Three healthy volunteers (average age: 28 years) were selected. Pathological abnormalities were excluded during the clinical examination of general and dental health. Other exclusion criteria were smoking, pregnancy or breastfeeding, the presence of dry mouth, taking medication or using mouthwash solutions such as CHX or antibiotics in the past three months. The study protocol was reviewed and approved by the local ethics committee (no. 502/13). An explanation and declaration of consent were signed by all participants in advance and are available in writing. In order to avoid interruptions in biofilm formation, the maxillary splints with the specimens were not cleaned during each period and were only removed and temporarily stored in sterile saline solution (0.9% NaCl) during meals and oral care. After biofilm formation, the BES were removed from the mouth and released from the splint. The specimens were then rinsed with 0.9% NaCl to remove non-adherent salivary remnants and bacteria before the biofilm was treated further [43].

### 2.3. Treatment and Cultivation of Biofilm Samples

After the natural biofilm formation, the BES with the adhering biofilm were placed in previously prepared solutions in multiwell cell culture plates (24-well multiwell plate, Greiner Bio-One, Frickenhausen, Germany) and incubated for two minutes in the dark to allow the photosensitizer to penetrate the biofilm without any influence of daylight. An effect by daylight may interfere with the effects of the novel light source consisting of VIS+wIRA. In addition to the concentrations of 300 and 450 µg/mL ICG to be tested, an untreated sample served as negative control (0.9% NaCl), while a sample treated with 0.2% chlorhexidine (CHX) served as positive control. The irradiation with VIS+wIRA was carried out for the samples with the photosensitizer for five minutes at 37 °C in a water bath, while the control groups stayed in the dark.

After treatment, the BES were transferred to sterile tubes (Eppendorf Tubes^®^, Eppendorf, Wesseling-Berzdorf, Germany) with 0.9% NaCl and were ultrasonicated for two minutes to remove the adherent bacteria of the initial (two hours) and mature biofilm (three days) from the enamel surface. The tubes were then vortexed using a vibration mixer (Vortex-Genie™ 2, Scientific Industries SI™, New York, NY, USA) for 30–45 s in order to release the microorganisms in the solution. The suspensions of the BES treated with aPDT, the untreated BES (negative control) and BES treated with CHX (positive control) were diluted according to a dilution series with 0.9% NaCl up to 1:10^4^ for the initial biofilm and up to 1:10^6^ for the mature biofilm. Each dilution was plated onto CBA and HCB plates followed by cultivation in an incubator (Heracell™ 150, Thermo Fisher Scientific Inc., Waltham, MA, USA) at 37 °C under aerobic conditions and anaerobic conditions using airtight reagent containers (Anaerocult^®^ A, Merck, Darmstadt, Germany). The subsequent quantification of the cultured microorganisms was carried out by visual determination of the CFU on the plates using a colony counter (WTW BZG 40, Xylem Analytics Germany Sales, Weilheim, Germany) and was part of a previous study [29].

### 2.4. Differentiation of the Microbial Composition

The bacterial colonies obtained after cultivation on the nutrient media were initially counted and differentiated according to their appearance, smell and hemolysis behavior. The creation of subcultures by means of a fractional three-eyelet smear was used to isolate noticeable bacteria in order to obtain pure cultures of the various microorganisms in the biofilm. The precise identification of the pure cultures was carried out using matrix-assisted laser desorption ionization-time-of-flight mass spectrometry (MALDI-TOF-MS) and was performed in a MALDI Biotyper (MALDI Biotyper Microflex LT, Bruker Daltonik, Bremen, Germany) already described previously [44]. According to the manufacturer’s information and settings, mass analysis spectra of the individual pure cultures were obtained which were compared using the Brucker Biotyper program (version 3.0, Bremen, Germany) with a reference database containing about 3740 reference mass analysis spectra. Resulting matches were given using a logarithmic scale value. An identification of the species corresponded to a value of ≥2.0, whereas a value of ≥1.7 only provided reliable information about the genus. At values below 1.7, there was no significant correspondence between the mass analysis spectrum obtained and the database entries. In the case of questionable results, the analysis of the bacterial culture was repeated several times until a clear assignment was possible.

### 2.5. Statistical Analysis

Due to the small sample numbers and massive detected microbial parameters, only a descriptive depiction of the results was performed.

## 3. Results

In the present study, the initial and mature supragingival biofilm was cultivated in vivo on bovine enamel slabs which were fixed on individual splint systems worn by three healthy volunteers. The oral biofilm was treated by visible light and water-filtered infrared A (VIS+wIRA) in combination with indocyanine green (ICG) and the surviving bacteria were isolated and identified using the culture technique and matrix-assisted laser desorption ionization-time-of-flight mass spectrometry (MALDI-TOF-MS). Untreated biofilm samples were used as negative control and biofilm samples treated with chlorhexidine (CHX) served as positive control.

### 3.1. Reduction in Bacterial Diversity within the Initial Biofilm after Treatment with VIS+wIRA in Combination with ICG as Photosensitizer 

By differentiating the microbial mass analysis spectra, individual bacterial species could be recorded and their proportion in the bacterial community determined. The comparison of the detected microorganisms in the untreated negative control with those of the positive control treated with CHX and the microbial composition of the biofilm treated the tested concentrations of ICG showed that there was a change in the oral biofilm composition. The percentages in the following explanation for the respective formation time refer to all examined subjects together.

**Figure 1:**
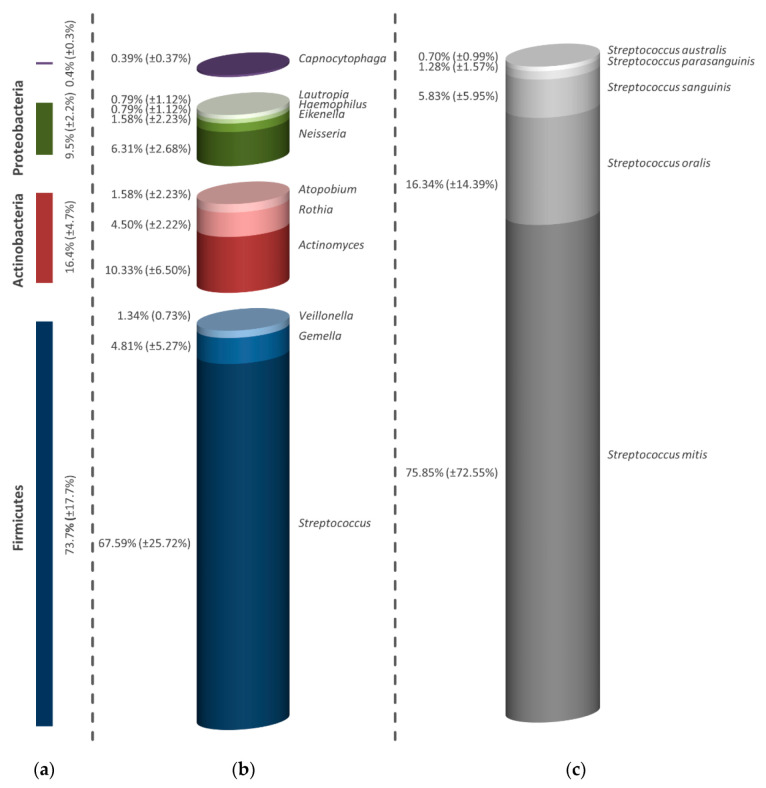
Column diagrams of the different microorganisms of the initial biofilm in the untreated control. The composition is shown in strain (**a**), genus (**b**) and bacterial species within the highest genus (**c**). All percentages given refer to all examined subjects. The bars depict the average values in percentage of three discs (one from each volunteer). In addition to the average values, the standard deviations are also presented in parentheses.

In the negative control of the initial biofilm after two hours, shown in Figure 1a, four different groups of bacteria were identified. Within Firmicutes, numerous streptococci (67.59%), *Gemella* spp. (4.81%) and *Veillonella* spp. (1.34%) were detected. In addition, Actinobacteria like *Actinomyces* spp. (10.33%), *Rothia* spp. (4.50%) and *Atopobium* spp. (1.58%), and the Proteobacteria *Neisseria* spp. (6.31%), *Eikenella* spp. (1.58%), *Haemophilus* spp. (0.79%) and *Lautropia* spp. (0.79%), were registered. Furthermore, *Capnocytophaga*, from the phylum of Bacteroidetes, was found to a small extent (0.39%) (Figure 1b). The evaluation of different streptococci in Figure 1c, which accounted for the largest percentage of initially adhering bacteria, shows a dominance of *Streptococcus mitis* with 75.85% over other streptococci such as *Streptococcus oralis* (16.34%), *Streptococcus sanguinis* (5.83%), *Streptococcus parasanguinis* (1.28%) and *Streptococcus australis* (0.70%).

**Figure 2:**
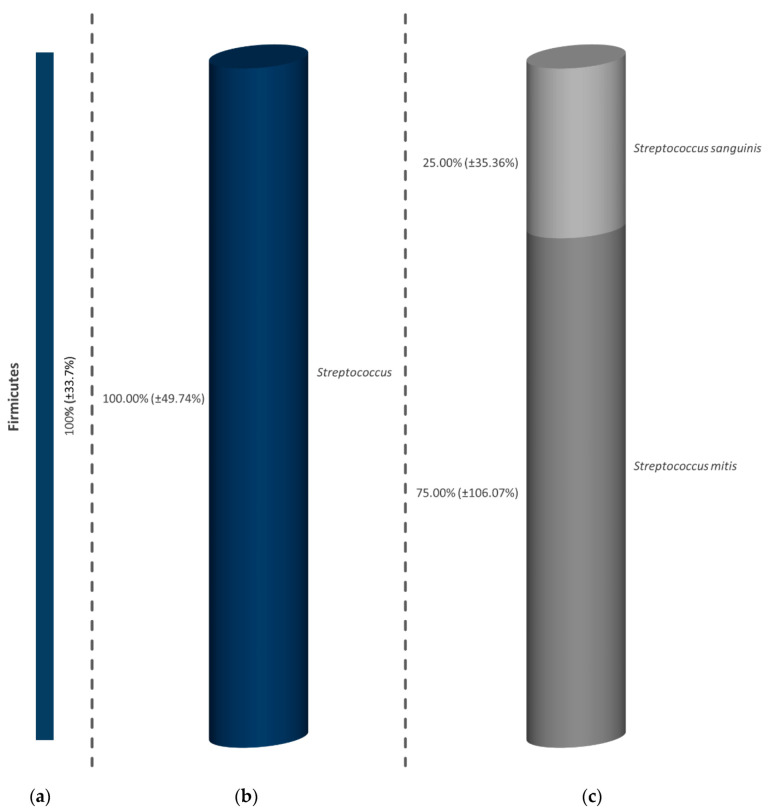
Graph of the surviving microorganisms after treatment of the initial biofilm with CHX. The subdivision is made into strain (**a**), genus (**b**) and bacterial species (**c**), whereby the occurrence is given in percentage, based on the total number of all detected bacteria in all subjects. The bars depict the average values in percentage of three discs (one from each volunteer). In addition to the average values, the standard deviations are also presented in parentheses. The high standard deviations are caused by the fact that bacteria were detected only in the initial adhesion of two volunteers after the treatment with CHX. All of these bacteria are members of the genus Streptococcus, of which Streptococcus mitis was detected only in the initial biofilm of one volunteer and Streptococcus sanguinis survived in the initial oral biofilm of two participants.

By using 0.2% CHX in the positive control, the composition of bacteria in all subjects was limited to Firmicutes (Figure 2a). As can be seen in Figure 2b, the diversity of 11 different bacterial genera in the negative control was reduced to streptococci (100.00%), whereby only *S. mitis* (75.00%) and *S. sanguinis* (25.00%) could be determined (Figure 2c).

**Figure 3:**
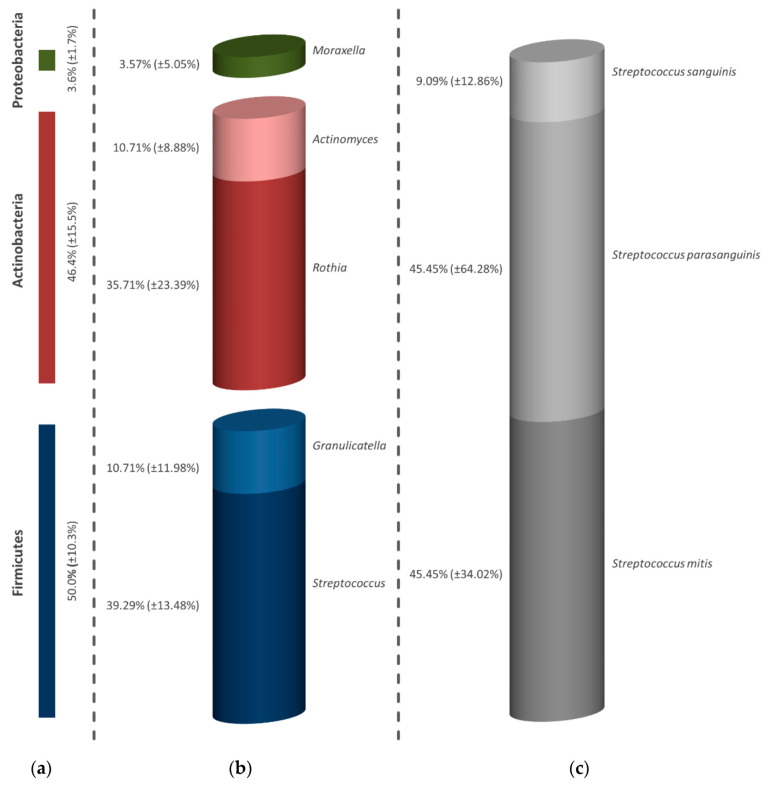
Presentation of the reduced diversity after application of aPDT in combination with 300 µg/mL ICG on the initial biofilm. The graph is divided into strain (**a**), genus (**b**) and bacterial species of the dominant genus of all examined subjects (**c**). The proportion of bacteria is given in percentages. The bars depict the average values in percentage of six discs (two from each volunteer). In addition to the average values, the standard deviations are also presented in parentheses.

In the samples treated with a concentration of 300 µg/mL ICG, a shift in the percentages of Firmicutes (50.0%), Actinobacteria (46.4%) and Proteobacteria (3.6%) was found compared to the untreated control, whereas representatives of Bacteroidetes could no longer be detected (Figure 3a). Further, also noticeable is the reduction in bacterial genera after the irradiation of the initial biofilm shown in Figure 3b, which were limited to *Streptococcus* spp. (39.29%), *Granulicatella* spp. (10.71%), *Rothia* spp. (35.71%), *Actinomyces* spp. (10.71%) and *Moraxella* spp. (3.57%). The barely predominant proportion of streptococci consisted equally of *S. mitis* (45.45%) and *S. parasanguinis* (45.45%) as well as a smaller proportion of *S. sanguinis* (9.09%) (Figure 3c).

In the case of the tested concentration of 450 µg/mL ICG, the photosensitizer as part of the aPDT was able to kill all pathogens in all examined subjects, so that no microorganisms could be identified.

### 3.2. Change in the Microbial Composition of Mature Oral Biofilm after Performing aPDT with ICG

**Figure 4:**
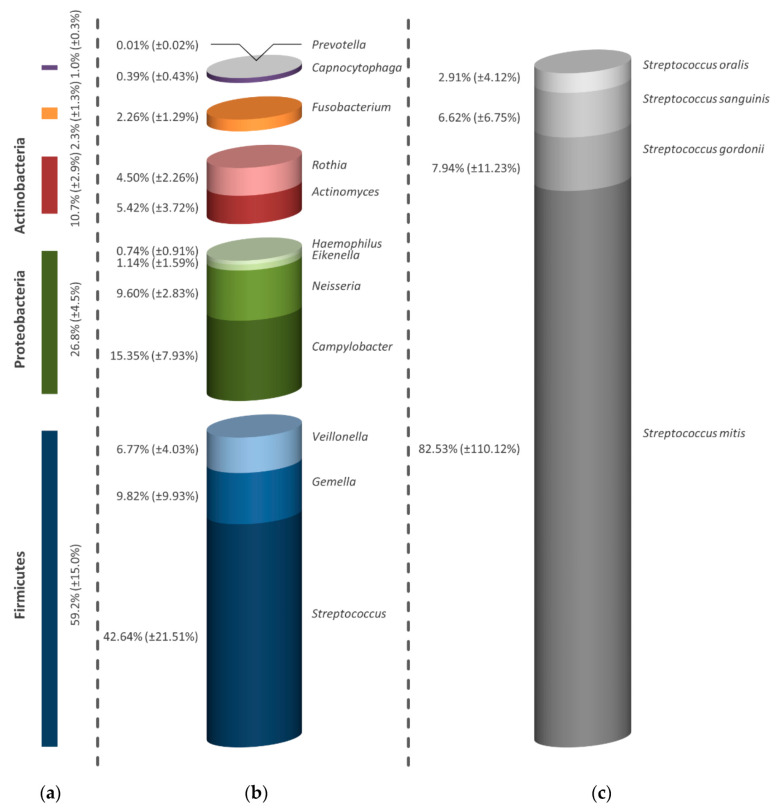
Diagrams illustrating the diversity of in situ generated biofilm samples after a formation time of 3 days. The depiction of the negative control shown is divided into strain (**a**), genus (**b**) and bacterial species of the most representative genus (**c**). All percentages shown refer to the total of the determined microorganisms in all subjects. The bars depict the average values in percentage of three discs (one from each volunteer). In addition to the average values, the standard deviations are also presented in parentheses.

In the evaluation of the untreated negative control of the mature oral biofilm, apart from Firmicutes, Proteobacteria and Actinobacteria, also Fusobacteria and representatives of Bacteroidetes could be distinguished (Figure 4a). Similar to the initial biofilm, streptococci represented the largest proportion of bacteria within the microbial composition of the mature oral biofilm with 42.64%, as shown in Figure 4b. With regard to the percentage distribution, *Campylobacter* spp. followed with 15.35% and with almost 10% *Gemella* spp. (9.82%) and *Neisseria* spp. (9.60%), which were also present in the biofilms. In addition, smaller proportions of *Veillonella* spp. (6.77%), *Eikenella* spp. (1.14%), *Haemophilus* spp. (0.74%), *Actinomyces* spp. (5.42%), *Rothia* spp. (4.50%), *Fusobacterium* spp. (2.26%), *Capnocytophaga* spp. (0.39%) and *Prevotella* spp. (0.01%) could be identified. Within the dominant genera of streptococci, *S. mitis* showed an especially high proportion of 82.53%. Further, proportions of *S. gordonii* (7.94%), *S. sanguinis* (6.62%) and *S. oralis* (2.91%) were also detected (Figure 4c).

**Figure 5:**
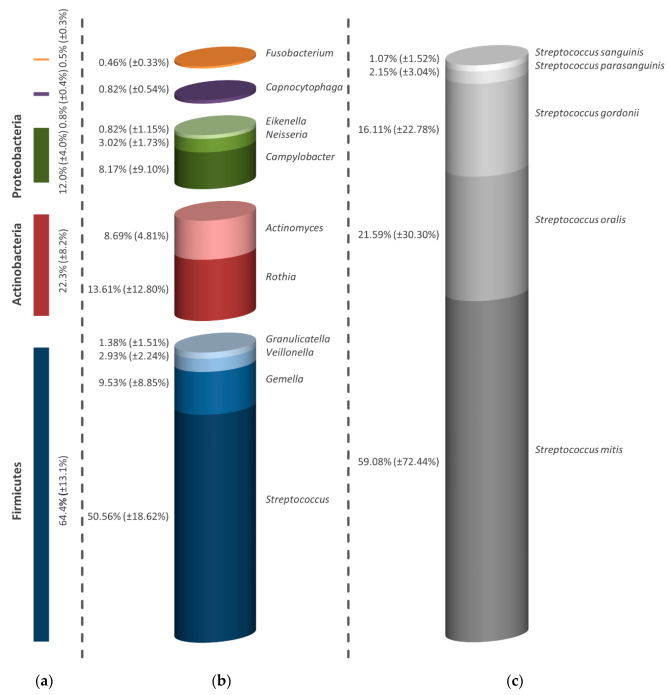
Illustration of the changed composition of the mature oral biofilm after treatment with 0.2% CHX. Division of diversity into bacterial strain (**a**), genus (**b**) and bacterial species of the proportionately largest genus (**c**). All determined percentages take into account the results of all examined subjects. The bars depict the average values in percentage of three discs (one from each volunteer). In addition to the average values, the standard deviations are also presented in parentheses.

The comparison of the positive control in Figure 5a and the negative control (Figure 4) shows that even after using CHX, Firmicutes (64.4%), Actinobacteria (22.3%), Proteobacteria (12.0%), Bacteroidetes (0.8%) and Fusobacteria (0.5%) could be found, whereby a shift in the respective percentages could be determined. Furthermore, the bacterial genera of the examined mature biofilm showed only a slight change in diversity, so that the genera *Streptococcus*, *Gemella*, *Veillonella*, *Granulicatella*, *Rothia*, *Actinomyces*, *Campylobacter*, *Neisseria*, *Eikenella*, *Capnocytophaga* and *Fusobacterium* could be detected (Figure 5b). More than half of the identified bacteria (50.56%) belonged to streptococci which were mainly represented by *S. mitis* (59.08%). Detected streptococci also include *S. oralis*, *S. gordonii*, *S. parasanguinis* and *S. sanguinis*, as shown in Figure 5c.

**Figure 6:**
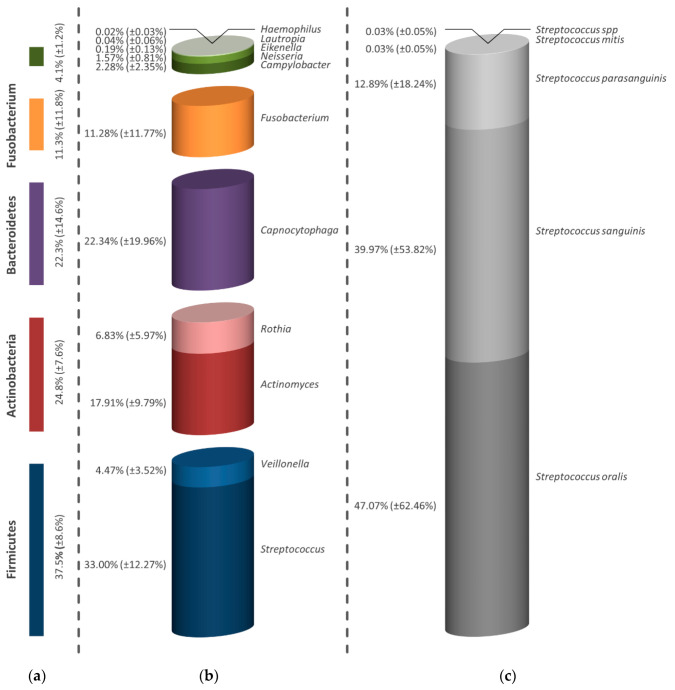
Graph of the microbial composition of the mature oral biofilm after application of aPDT in combination with 300 µg/mL ICG. The percentages shown are related to total samples divided into strain (**a**), genus (**b**) and bacterial species of the dominant genus (**c**). The bars depict the average values in percentage of six discs (two from each volunteer). In addition to the average values, the standard deviations are also presented in parentheses.

According to the illustration of the biofilm treated with 300 µg/mL ICG shown in Figure 6a, five different bacterial strains were still present. In addition to a reduced proportion of Firmicutes (37.5%) and Proteobacteria (4.1%), a particularly high proportion of Bacteroidetes (22.3%) and Fusobacteria (11.3%) was noticeable compared to the negative control (Figure 4). The detected bacterial genera in Figure 6b, which are *Streptococcus*, *Veillonella*, *Actinomyces*, *Rothia*, *Capnocytophaga*, *Fusobacterium*, *Campylobacter*, *Neisseria*, *Eikenella*, *Lautropia* and *Haemophilus*, only suggest a slight reduction in diversity. Although streptococci represented a smaller proportion of the microbial composition compared to the untreated control, these microorganisms formed the largest genus (33.00%) within the biofilm samples treated with ICG. The identified streptococci consisted of *S. oralis* (47.07%), *S. sanguinis* (39.97%), *S. parasanguinis* (12.89%), *S. mitis* (0.03%) and *Streptococcus* spp. (0.03%) (Figure 6c).

**Figure 7:**
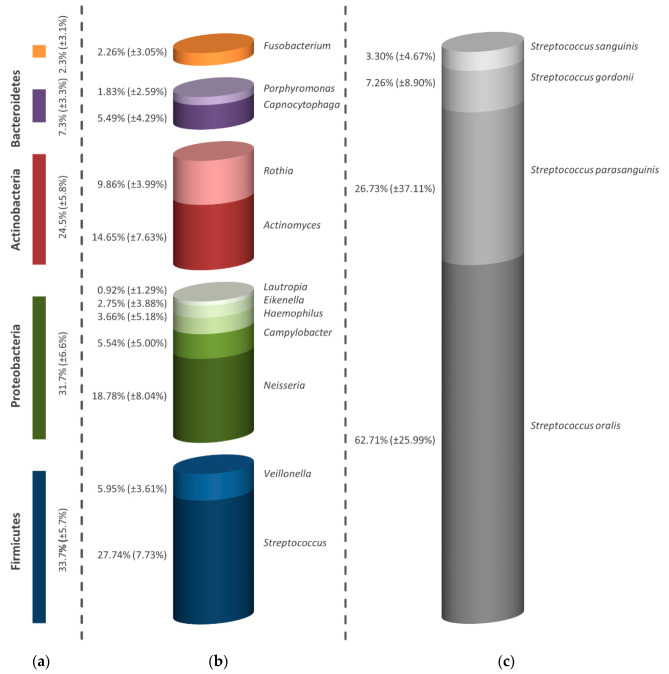
Visualization of surviving microorganisms within the mature biofilm treated with 450 µg/mL ICG after aPDT. The percentages of the bacteria in the total microbial composition of all examined subjects are given. The graph shows a division into strain (**a**), genus (**b**) and dominant bacterial species (**c**). The bars depict the average values in percentage of six discs (two from each volunteer). In addition to the average values, the standard deviations are also presented in parentheses.

Even after treatment with aPDT and a higher concentration of 450 µg/mL ICG, Firmicutes, Proteobacteria, Actinobacteria, Bacteroidetes and Fusobacteria could be detected, as shown in Figure 7a. In comparison with the untreated control, the percentage of Firmicutes was reduced to 33.7% with the help of the tested concentration, leading to an increase in the other percentages. However, no clear reduction in the number of bacterial genera was found (Figure 7b). Apart from a reduced proportion of streptococci (27.74%), *Veillonella* spp. (5.95%), *Neisseria* spp. (18.78%), *Campylobacter* spp. (5.54%), *Haemophilus* spp. (3.66%), *Eikenella* spp. (2.75%), *Lautropia* spp. (0.92%), *Actinomyces* spp. (14.65%), *Rothia* spp. (9.86%), *Capnocytophaga* spp. (5.49%), *Porphyromonas* spp. (1.83%) and *Fusobacterium* spp. (2.26%) could also be classified. As can be seen in Figure 7c, the streptococci were largely composed of *S. oralis* (62.71%), but also of *S. parasanguinis* (26.73%), *S. gordonii* (7.26%) and *S. sanguinis* (3.30%).

## 4. Discussion

Microorganisms within biofilms are highly resistant towards antibiotics and disinfectants [45,46]. Hence, complementary procedures, such as aPDT, offer a good opportunity to eradicate bacteria also within the oral biofilm [23,38,47]. Based on promising results with other photosensitizers, the effect of aPDT using VIS+wIRA in combination with ICG on oral biofilm composition was examined after formation periods of two hours and three days [28]. In contrast to the other studies with ICG, the biofilm examined was generated in situ on bovine enamel slabs and subjected to aPDT ex vivo. A previous study showed that VIS+wIRA in combination with ICG significantly reduced the number of planktonic bacteria within human saliva as well as the viable counts of oral microorganisms within initial adhesion and mature oral biofilms [29]. In the present study, the aim was to investigate whether aPDT with VIS+wIRA as the light source and ICG as the photosensitizer is able to change the diversity of living bacteria in the biofilm and to influence their microbial balance.

In terms of density, mineralization and structure, bovine enamel slabs (BES) show almost identical physicochemical properties to human enamel samples and are therefore well suited for the generation of biofilms in situ [42,48,49]. Due to these advantages, BES were used in this study.

Unlike most previous experiments with ICG, broadband heat radiation filtered by a water band was used in this study. The halogen lamp of the radiator produces a wide emission wavelength spectrum in the range of 570–1400 nm, which can be seen as an advantage over conventional LED and laser light sources with a limited wavelength spectrum. The additional water filtering largely reduces the proportion of released infrared B and infrared C in order to reduce unwanted skin damage and to prevent overheating as caused by conventional halogen lamps [25,50,51]. An increase in the temperature of the tooth pulp was also observed when using LED lamps, which can be assessed as a disadvantage [52]. Due to their high penetration capacity by VIS+wIRA, microorganisms can be affected even in deeper tissue layers, characterized by a reduced effect of antibiotics under oxygen deficiency in the subcutaneous tissue [53]. Additionally, the penetration of VIS+wIRA in deeper tissue layers provides an opportunity to reduce bacteria in the poorly accessible biofilm. The depth effect of VIS+wIRA is also evident in an increased tissue perfusion, a temperature rise of 2.7 °C in a tissue depth of 2 cm and an increase in the prevailing oxygen partial pressure by 30% [53,54,55]. The resulting activation of the immune system and promotion of wound healing are a potential positive aspect, especially with regard to the treatment of periodontitis or peri-implantitis, which are based on an inflammatory process with locally weakened immune defense [56]. In addition, the increased tissue blood flow enables the elimination of bacterial toxins and improves the local defense against infection in the affected tissue via subsequent immunomodulation effects [25]. The irradiation time in the present study was set to five minutes in order to simulate a realistic duration of use in daily dental practice with regard to an adjunctive treatment.

In contrast to other dyes, ICG, as a representative of the polymethine group, has been approved by the Food and Drug Administration (FDA) and shows differences in its effects compared to established photosensitizers [57,58]. Since most of the absorbed light is converted into fluorescence and heat during application, ICG can be assumed to mainly have a photothermic effect in combination with a photodynamic effect [18,59]. In oncology, in addition to the fluorescence of the photosensitizer, the combined effects of the formation of reactive oxygen species and the hyperthermia effect of ICG are used to detect tumors and to eliminate them in a minimally invasive manner [60]. A substance-removing effect of pathologically altered tissue could also be demonstrated in cariology through the use of aPDT in combination with ICG [61,62]. Due to the nonspecific reaction of ICG-based aPDT, heat-induced tissue damage is possible, so that a suitable irradiance and duration of use should not be exceeded [63]. Using ICG with a low laser power (200–400 mW), on the other hand, might be gentle on the tissue and, in addition to microbial elimination including antibiotic-resistant microorganisms, could lead to an improvement in wound healing [32].

After carrying out the aPDT and successfully cultivating surviving microorganisms, visible bacterial colonies were classified according to morphological and biochemical criteria and transferred to pure cultures. Due to the cultivation, difficulties arose in differentiating the total microbial population of the biofilm. Different nutrient requirements and growth rates of the different bacterial species, which can compete with each other, only allow a smaller part of the bacterial composition to be recorded with this method, since not all bacterial species are cultivable and thus visible on the nutrient media [64]. According to the literature, it is not possible to cultivate the majority of the microorganisms that colonize the oral cavity [65]. Within the limits of this method, MALDI-TOF-MS was used to precisely identify cultivable bacteria. The method was characterized by its ease of use, short analysis times and high precision at a relatively low cost [44]. Mass spectrometry can also reliably identify all bacteria stored in databases, independent of the nutrient media, and is able to replace time-consuming biochemical differentiation methods for routine use [66,67]. The identification of bacteria using biochemical methods requires an additional 48 h after obtaining the pure bacterial cultures compared to the five minutes analysis time of pure bacterial isolates using MALDI-TOF-MS [44]. Furthermore, the comparison of MALDI-TOF-MS with the 16S rDNA sequencing method, considered the gold standard in bacterial identification, revealed the excellent discriminatory power of MALDI-TOF-MS to differentiate critical oral lactobacillus species, which have substantial similarities in their 16S rDNA genes [44].

In addition to the bacteria-eliminating effect of ICG reported in an earlier study, the influence of aPDT on the composition of the oral biofilm could be also meaningful for the adjunctive treatment of periodontitis and peri-implantitis [3,4,5,29]. In the untreated initial biofilm, numerous bacterial species were identified, and can be summarized in 11 bacterial genera. These microorganisms mainly belonged to the early colonization group [68]. At almost 68%, oral streptococci were the predominant microorganisms of the initial biofilm. This result is also reflected in the literature, which describes a biofilm with an oral streptococcal fraction of 60–90% a few hours after cleaning the tooth surfaces [69].

Treatment with CHX (0.2%) almost completely killed all microorganisms with the exception of a few streptococci, which confirms the bactericidal effect of the concentration used [70]. The initial biofilm was shown to be more easily eradicated by CHX than the mature biofilm. This is in agreement with already described results in the literature [71]. As a possible explanation, the higher susceptibility of Gram-positive bacteria, which form the majority of the microorganisms of the initial biofilm, to CHX compared to Gram-negative bacteria can be considered [72].

In all subjects, a high reduction in diversity at a lower ICG concentration and the complete elimination of all microorganisms at a higher ICG concentration was achieved by aPDT. The results of a high reduction in diversity show the effectiveness of aPDT with ICG, especially at the beginning of biofilm formation. This could be due to the fact that the initial biofilm is still unstable and therefore susceptible to antimicrobial substances [73,74]. The reduction in the number of bacteria and the low diversity of the initial biofilm hinder further biofilm formation, as the lack of early colonizers means that biofilm formation could be disturbed or only partially possible [75].

In contrast to the initial adhesion, the treatment of the mature oral biofilm was characterized by the presence of numerous other bacterial species which could be classified into 12 different bacterial genera. Oral streptococci also accounted for the largest proportion of the total bacterial count in this stable bacterial network. Unlike the microbial composition of the initial biofilm, late colonizers were also found, such as *Campylobacter* spp., *Capnocytophaga* spp., *Veillonella* spp. and *Fusobacterium* spp., which have an important function as bridging agents within the biofilm [76,77].

In the mature oral biofilm, treatment with CHX showed only a reduction in diversity to 11 different bacterial genera. This result emphasizes the higher resistance of the microorganisms within the mature biofilm compared to the initially adhered microorganisms, which apart from a few streptococci were eradicated. This increased tolerance of microorganisms growing in biofilms to antimicrobial agents is attributed to the presence of a biofilm matrix, also known as an extracellular polymeric substance (EPS), which due to its high viscosity can slow down drug diffusion or even act as a barrier. Additionally, an altered gene expression from local biofilm bacteria compared to planktonic cells is suspected [13].

A similarly small change in the composition was achieved with the tested ICG concentrations, which was mainly based on a shift in the percentages of the total microbial composition. According to these changes, the decline in Firmicutes is particularly noticeable, with Gram-positive bacteria such as *Gemella* spp. and *Streptococcus* spp. in particular decreasing. Primary colonizers as oral streptococci are essential for further biofilm formation, since late colonizers can only indirectly bind to the pellicle via these microorganisms [78]. Therefore, a reduction in these species leads to disturbances of the structural balance within the biofilm and makes it more difficult for further microorganisms to attach. Bacteroidetes, which belong to the Gram-negative bacteria, have been less reduced by ICG, which increases their proportion within the irradiated biofilm. This suggests that Gram-positive bacteria in particular are highly susceptible to aPDT with ICG, similar to the experience with CHX [72]. Although individual bacterial genera could be completely eliminated by treatment with the photosensitizer, the diversity of the biofilm was not changed or only changed slightly. Both the initial adhesion and the mature biofilm showed a heterogeneous composition of surviving microorganisms in the individual test subjects. This could mainly be attributed to individual saliva compositions and the different microbiological compositions of the individual participants before treatment. There are different reports regarding certain bacteria such as so-called mutans streptococci, consisting of *Streptococcus mutans* and *Streptococcus sobrinus*, which should be considered pathogenic in the supragingival oral biofilm regarding the development of caries. However, the ecological caries hypothesis and the extended ecological caries hypothesis consider a wide range of bacteria within the oral biofilm as the etiological cause of caries [79]. This means a balance within the total supragingival oral biofilm can be considered as pathogenic if exposed to a certain diet containing a high proportion of carbohydrates like sugar. Hence, an alteration of biofilm diversity by aPDT may reduce its pathogenicity.

## 5. Conclusions

The treatment of the initial and mature biofilm with VIS+wIRA in combination with ICG causes a change in the biofilm composition in addition to the primary reduction in the number of living bacteria. Gram-positive microorganisms appear to be more sensitive to treatment with VIS+wIRA and ICG. Hence, the balance of microorganisms within the oral biofilm seems to be disturbed by this novel aPDT applied here. This can be considered as a successful adjunctive treatment method for peri-implantitis and periodontitis and can increase the susceptibility of the oral biofilm to further therapeutic interventions involving conventional antimicrobials. The clinical impact of this novel approach should be evaluated in studies involving dental patients suffering from periodontitis or peri-implantitis.
